# 2709. Breakthrough fungal infection after isavuconazole primary prophylaxis in patients with hematologic malignancy and hematopoietic stem cell transplant: Systematic Review

**DOI:** 10.1093/ofid/ofad500.2320

**Published:** 2023-11-27

**Authors:** Keiko Ishida, Mizuki Haraguchi, Muneyoshi Kimura, Hideki Araoka, John M Reynolds, Mohammed Raja, Yoichiro Natori

**Affiliations:** Toranomon Hospital, Niijyuku, Katushikaku, Tokyo, Japan; Toranomon Hospital, Niijyuku, Katushikaku, Tokyo, Japan; Toranomon Hospital, Niijyuku, Katushikaku, Tokyo, Japan; Toranomon Hospital, Niijyuku, Katushikaku, Tokyo, Japan; Louis Calder Memorial Library/University of Miami, Miami, Florida; University of Miami Miller School of Medicine/Sylvester Comprehensive Cancer Center, Miami, Florida; University of Miami, Miami Transplant Institute, Jackson Health System, Miami, Florida

## Abstract

**Background:**

Isavuconazole (ISA) is a relatively newer triazole with a broad spectrum of anti-fungal activity and has been used for both treatment and prophylaxis of invasive fungal infections (IFIs) in patients with hematologic malignancy (HM) and hematopoietic stem cell transplant (HSCT) with a more favorable side-effect profile as compared to other agents. Widespread prophylactic use of mold-active agents has resulted in a decline in the incidence of IFIs, but breakthrough (bIFIs) has been reported previously. Thus, we performed a systematic review of HM and HSCT patients receiving ISA as primary prophylaxis to determine incidence and clinical characteristics of bIFI in this vulnerable population.

**Methods:**

We conducted a comprehensive literature search with search term of ISA, prophylaxis and either HM and/or HSCT in several search engines (Embase, Scopus Web of Science, MEDLINE and CENTRAL). The database search strategy was developed by an academic health science librarian. We excluded patients who had ISA as treatment or secondary prophylaxis.

**Results:**

With initial search, 630 unique articles were identified and after reviewing abstracts, we finally conducted a full-text review of 70 articles, of which 27 studies were included (Figure), of which 17 studies included more than or equal to 10 patients on ISA prophylaxis. Overall, ISA was well tolerated during primary prophylaxis. Of note, of the 17 studies, 717 patients received ISA as primary prophylaxis, out of which 58 patients (8.1%) developed bIFI with range of 0%-20% and occurred 13-138 days after initiation (Table). ISA levels were checked in 6 studies with a plasma level between 1.5-6.3 μg/mL. Out of 58 bIFI cases, 33 cases were identified including 6 Mucorales spp., 3 Fusarium spp., 8 Candida spp and 14 Aspergillus spp. Most commonly used regimen for treatment of bIFI was combination of Liposomal amphotericin B with Posaconazole. Overall mortality after bIFI was 0%-66.7%.

**Figure:**
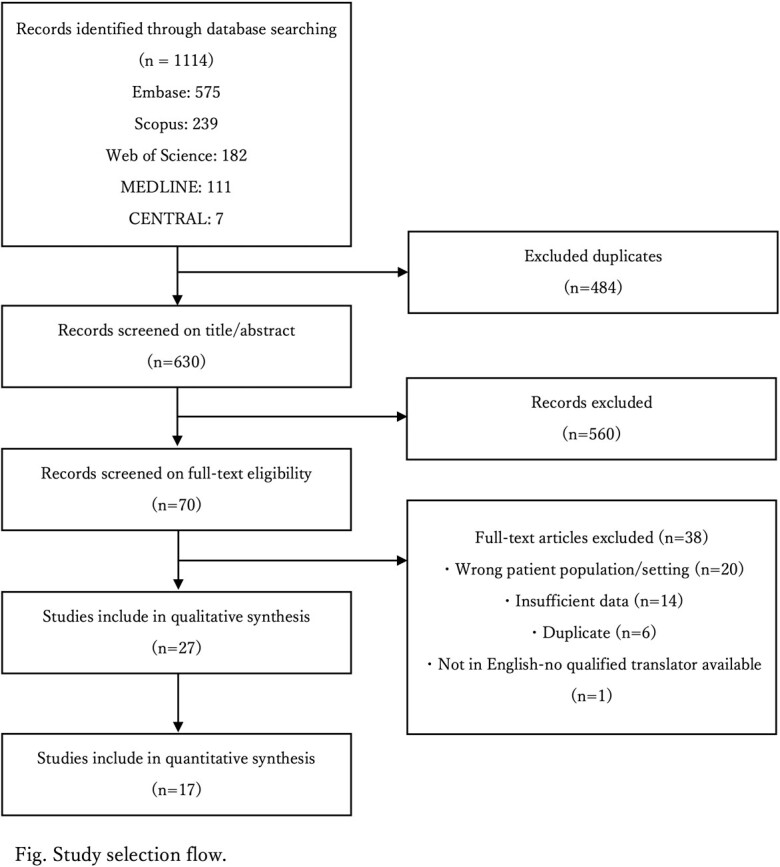

**Figure:**
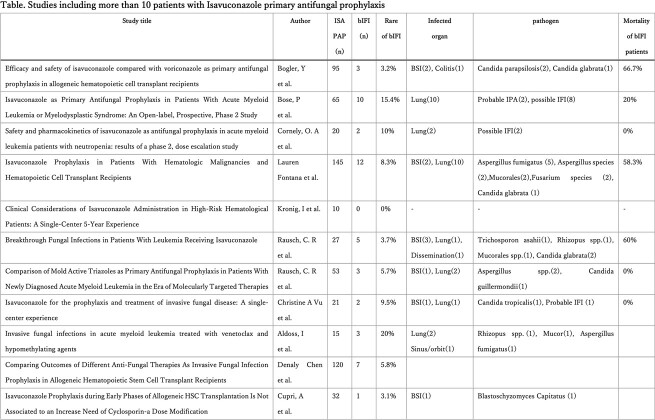

**Figure:**
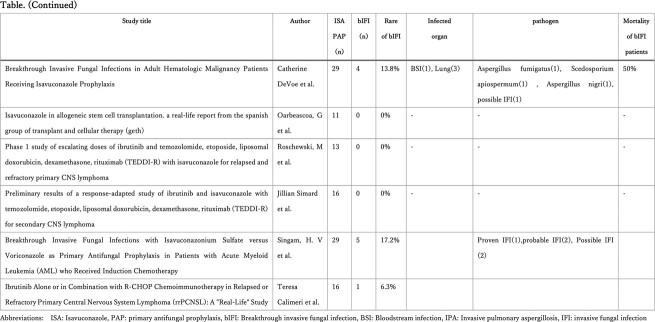

**Conclusion:**

Our systematic review shows a high incidence of bIFI in patients with underlying HM or HSCT receiving ISA prophylaxis. Caution should be used in patients receiving long term prophylaxis with ISA. Further studies are needed to assess for risk factors that predispose to bIFI in patients receiving ISA prophylaxis.

**Disclosures:**

**John M. Reynolds, MLIS**, Pfizer Inc: Stocks/Bonds

